# Intestinal Microbiota Distinguish Gout Patients from Healthy Humans

**DOI:** 10.1038/srep20602

**Published:** 2016-02-08

**Authors:** Zhuang Guo, Jiachao Zhang, Zhanli Wang, Kay Ying Ang, Shi Huang, Qiangchuan Hou, Xiaoquan Su, Jianmin Qiao, Yi Zheng, Lifeng Wang, Eileen Koh, Ho Danliang, Jian Xu, Yuan Kun Lee, Heping Zhang

**Affiliations:** 1 Key Laboratory of Dairy Biotechnology and Bioengineering, Education Ministry of P. R. China, Huhhot, Inner Mongolia, 010018, China; 2 Department of Microbiology, Yong Loo Li School of Medicine, National University of Singapore, 5 Science Drive 2, 117597, Singapore; 3 The First Affiliated Hospital, Baotou Medical College, Baotou, Inner Mongolia, 014010, China; 4 Single-Cell Center, Qingdao Institute of Bioenergy and Bioprocess Technology, Chinese Academy of Sciences, Qingdao, Shandong, 266101, China

## Abstract

Current blood-based approach for gout diagnosis can be of low sensitivity and hysteretic. Here via a 68-member cohort of 33 healthy and 35 diseased individuals, we reported that the intestinal microbiota of gout patients are highly distinct from healthy individuals in both organismal and functional structures. In gout, *Bacteroides caccae* and *Bacteroides xylanisolvens* are enriched yet *Faecalibacterium prausnitzii* and *Bifidobacterium pseudocatenulatum* depleted. The established reference microbial gene catalogue for gout revealed disorder in purine degradation and butyric acid biosynthesis in gout patients. In an additional 15-member validation-group, a diagnosis model via 17 gout-associated bacteria reached 88.9% accuracy, higher than the blood-uric-acid based approach. Intestinal microbiota of gout are more similar to those of type-2 diabetes than to liver cirrhosis, whereas depletion of *Faecalibacterium prausnitzii* and reduced butyrate biosynthesis are shared in each of the metabolic syndromes. Thus the Microbial Index of Gout was proposed as a novel, sensitive and non-invasive strategy for diagnosing gout via fecal microbiota.

Gout is an auto-inflammatory disease caused by a disorder in purine metabolism and the resulted chronic elevation of blood uric acid (i.e., hyperuricemia)^1^. With increased intake of high protein food in many societies, incidents of gout has been expanding at an alarm rate worldwide^2^. In 2011, prevalence of gout in US adults is about 3.9%, and that of hyperuricemia which is a precondition for developing gout reached up to 21%^3^. In UK, prevalence of gout has risen to 2.5% of the general population in 2012, an increase of 63.9% since 1997^4^. In China, gout was previously extremely rare, yet the number of confirmed cases has reached 75 million by the end of 2010^5^.

Despite the expanding prevalence of the disease, accurate diagnosis remains a challenge. Pathogenesis of gout is closely related to the increased accumulation and the reduced excretion of uric acid (the end product of purine metabolism). The resulted deposition of uric acid salt crystals in joints and the surrounding tissues lead to acute joint pain^6^. Therefore, the two symptoms of (*i*) deposition of uric acid salt combined with acute pain in and around joints and (*ii*) increase in uric acid level in blood, are presently the clinical diagnostic criteria for gout^7^. The latter, termed the blood uric acid value, is considered as the major diagnostic criteria in current clinical practice, as it is quantifiable. However the blood uric acid index seems to be hysteretic in a large part and not sufficiently sensitive^8^. Individuals suffered from gout are frequently in a stressed state, which causes reflective secretion of adrenocorticotrophic hormone to compel the discharge of uric acid by kidney^9^. Consequently, there can be no significant rise of uric acid for most early-onset gout patients^8^. Therefore method development for early diagnosis of gout has been of high priority.

Healthy humans excrete uric acid in two main ways, with 70% excreted through the kidney and the remaining 30% via the intestine^10^. Human intestine homes a huge number of microbes collectively known as intestinal microbiota, whose activities are linked to numerous host functions^11^,^12^,^13^,^14^,^15^. The intestinal microbiota are known to participate in metabolism of purine and uric acid. For example, in the oxidative metabolism of purine, the responsible enzyme xanthine dehydrogenase can be secreted by the *Escherichia coli* group of human intestinal bacteria^16^,^17^. In uric acid catabolism, uricase, allantoinase and allantoicase activities can sequentially degrade uric acid to 5-hydroxyisourate, allantoin, allantoate and eventually to urea. Synthesis of these enzymes was found vigorous in *Lactobacillus* and *Pseudomonas*, both common members of human intestinal microbiota^18^. Moreover transport protein of uric acid were found secreted by various indigenous microbes in human gut^19^. Therefore, we hypothesize that the intestinal microbiota can potentially serve as proxy to probe uric acid metabolism in the host for the purpose of diagnosis or prognosis.

To test this hypothesis, here we designed a cross-sectional study in an 83-member Chinese cohort that consists of both gout patients and healthy individuals. The taxonomic structure of their intestinal microbiota was determined by 16S rRNA gene pyrosequencing, and the functional profile of the corresponding microbiome was determined by shotgun metagenomic sequencing. Profound difference in microbial taxonomic and functional features was discovered between gout patients and healthy individuals. A microbiota-based model based on such difference was established and shown to diagnose gout at 88.9% accuracy.

## Results

### Intestinal microbiota altered profoundly in gout patients

The study cohort includes 83 Chinese adults (Table 1, Supplementary Table 1). The gout group consists of 35 adult patients who were clinically diagnosed by endocrinologists as gout based on blood uric acids, claim of joint pains and other parameters. The healthy group which serves as control consists of 33 healthy adults. An additional, 15-member group for model validation purpose consists of 6 gout patients and 9 healthy individuals. For each of the collectively 83 subjects, organismal structure of intestinal microbiota was analyzed by sequencing 16S rRNA gene amplicons, which revealed for each microbiota on average 202 operational taxonomic units (OTUs) from averagely 6402 reads (Supplementary Table 2). To characterize the functional profiles of microbiota, 16 from the gout group, 18 from the healthy group and 5 from the validation group were randomly selected for whole-metagenome shotgun sequencing, yielding 371.2 Giga base (Gb) of pair-end reads (averagely 59,681,612 high-quality reads for each microbiota; Supplementary Table 3).

**Figure 1:**
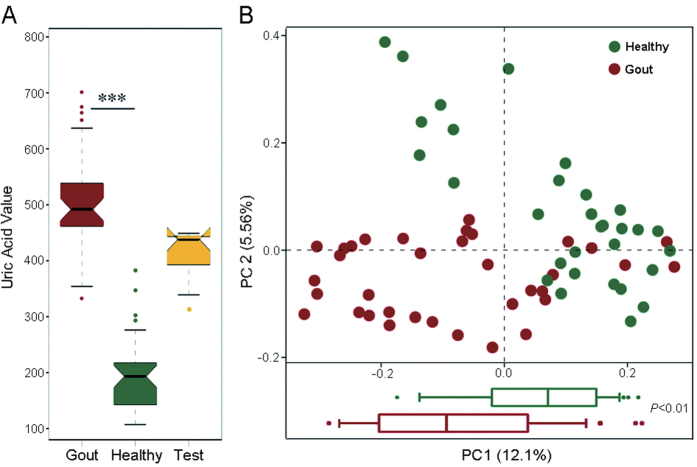
The composition of gut microbiota alters profoundly in gout patients. (**A**) The uric acid values in the gout patients, healthy (control) and validation groups. (**B**) A principal component (PCoA) score plot based on weighted UniFrac metrics for all participants. Each point represents the composition of the intestinal microbiota of one participant.

Between the gout group and the healthy group, no significant difference was found for the factors of age, gender or BMI. However, among the array of blood indices, significant differences (*P* < 0.001, Wilcoxon rank-sum test) were detected between the two groups in blood uric acid value, total bilirubin, glutamic-pyruvic transaminase (GPT), glutamic oxalacetic transaminase (GOT) and urea nitrogen level (Fig. 1A; Supplementary Table 1). Intriguingly, in the gout group, the blood uric acid values of patients 14, 27, 39 and 60 were lower than other patients, which is consistent with the notion of insufficient sensitivity of the blood uric acid index for diagnosing gout in certain patients^8^.

To test whether any difference in organismal structure of intestinal microbiota was present, Principal Coordinates Analysis (PCoA) was performed based on the weighted Unifrac distances of 16S rRNA sequence profiles at the genus level. A significant reduction was observed in α diversity of intestinal microbiota from the gout group as compared to those from the healthy group (*P* < 0.01, Wilcoxon rank-sum test; Supplementary Table 4), suggesting that a lower microbial diversity in the intestine was associated with gout. Moreover, intestinal microbiota from the gout group and the healthy group were highly distinct in organismal structure, with the subjects forming two clusters that respectively corresponded to the two groups (Fig. 1B, upper panel), and significant separation (*P* < 0.001) in PC 1 was observed (Fig. 1B, lower panel; Wilcoxon rank-sum tests).

To correct for any possible population stratification caused by the non-gout-related factors such as age, gender and BMI, organismal structures of the microbiota were further analysed using Permutational Multivariate Analysis of Variance (PERMANOVA). After the correction those effects correlated with the non-gout-related factors disappeared (Supplementary Table 5). This further confirmed that the gout disease is one significant factor in explaining the observed variation in organismal structure of intestinal microbiota.

Furthermore, to probe the difference in functional profile of intestinal microbiota between gout patients and healthy subjects, high-quality reads from all samples were assembled and annotated for protein-coding genes, based on which a collective, non-redundant intestinal microbiota gene catalog for gout was created. Then for each of the sample, its reads were mapped to the collective gene catalog to reconstruct sample-specific gene profile, as well as the profiles for the associated Clusters of Orthologous Groups (COG, Supplementary Table 6) and Kyoto Encyclopedia of Genes and Genomes database Orthologues (KO; Supplementary Table 7). For the gene profiles, PCA analysis revealed that two clusters that respectively correspond to the gout group and the healthy group were formed (Supplementary Fig. 1A), and the separation in PCA 1 between the two groups was significant (*P* < 0.001; pairwise Wilcoxon tests). These results suggested a distinct functional gene structure of the gout patients as compared to that of the healthy subjects.

To test the degree of consistency between the observed clustering patterns for organismal structure and functional structure respectively, Procrustes analysis was performed based on the PCA matrix of genus-level organismal profile and that of functional gene profile (Supplementary Fig. 1B). The results revealed a strong correspondence between microbial taxonomy and function (*P* < 0.001, using 10,000 Monte Carlo label permutations). Thus the classification of the samples along the line of the gout group of subjects and the healthy group is robust, and moreover, both organismal structure and functional gene structure of intestinal microbiota from an individual host carry significant and consistent information for the gout disease state.

### Microbial index of Gout

**Figure 2:**
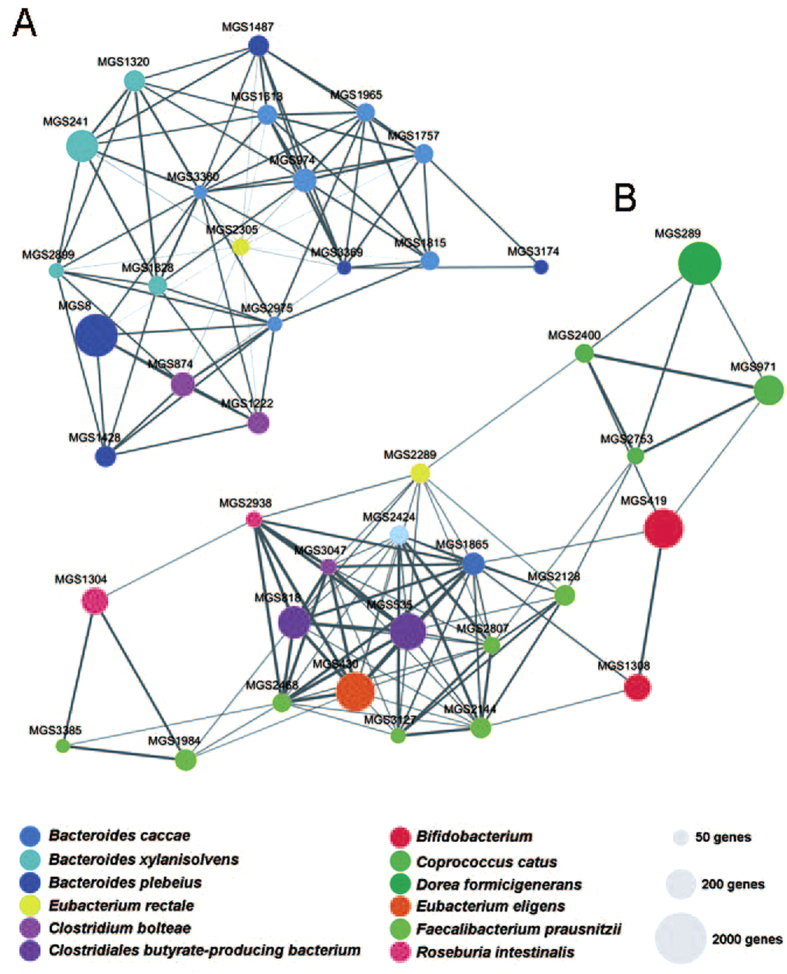
Taxonomic characterization of the intestinal microbiota in gout. Differentially abundant MGS networks enriched in gout patients (*n* = 19, panel A) and healthy individuals (*n* = 22, panel B). The edge width is proportional to the correlation strength. The node size is proportional to the mean abundance in the respective population. Nodes with the same color are classified in the same phylogenetic species. Every node represented a MGS.

To explore microbial species associated with gout disease, microbial genes predicted from the whole-metagenome sequencing data were grouped into clusters based on the gene abundance profiles, with each of the clusters denoted a metagenomic species (MGS)^20^. Among the 41 MGS identified from metagenomic species profile, 22 were enriched in healthy individuals, such as those MGS representing *Faecalibacterium prausnitzii, Clostridium* butyrate-producing bacterium and *Bifidobacterium pseudocatenulatum* (Wilcoxon rank-sum test; Fig. 2; Supplementary Table 8). On the other hand, 19 MGS were enriched in the gout patients, including species such as *Bacteroides caccae* and *Bacteroides xylanisolvens* (Fig. 2; Supplementary Table 8).

The highly distinct organismal features of gout suggested the possibility of classifying gout subjects based on the intestinal microbiota. On the other hand, based on the bacterial 16S rRNA pyrosequencing data, bacterial genera that were differentially distributed between the gout group and the healthy group were revealed. A total of 17 genera (*P* < 0.05, Wilcoxon rank-sum test) were associated with gout disease, with *Bacteroides, Holdemania, Anaerotruncus* and several others positively associated with the gout, whereas *Faecalibacterium, Coprococcus, Ruminococcus* etc negatively associated with the gout (i.e., they were enriched in the healthy group; Table 2). These results were consistent with the organismal features identified via the MGS analysis above.

To test this hypothesis of microbiota-based diagnosis of gout, the 68-host cohort that includes the 33-member gout group and the 35-member healthy group was used as a training set for model construction. A ‘microbial index of gout’ (MiG) was derived based on the relative abundance of the 17 bacterial markers that distinguish between the healthy and the gout patients in the 68-host cohort:


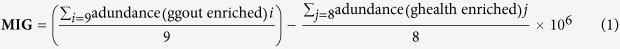


**Figure 3:**
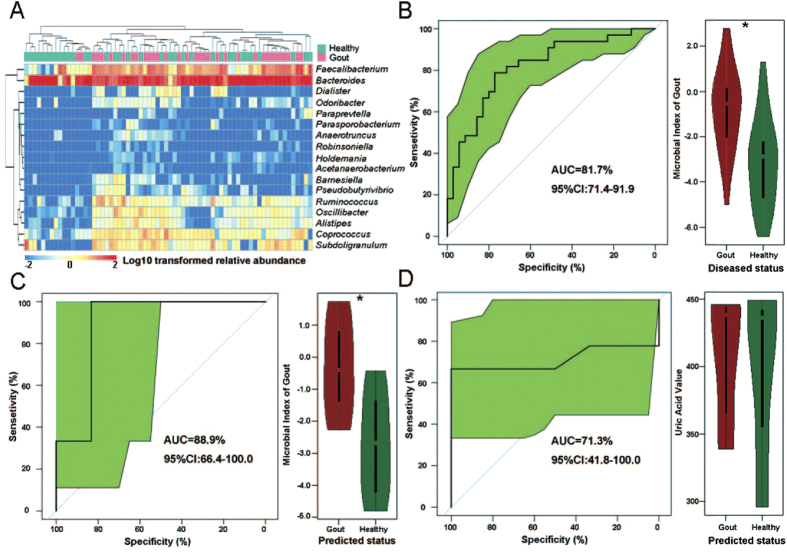
Classification of the gout status using bacterial genus-level biomarkers based on 16S pyrosequencing data and stratification of RISK hosts in a validation cohort. (**A**) The heatmap indicated the ability of the genus-level biomarkers to discriminate the healthy and gout groups. (**B**) Accuracy of the microbiota-based predictive model is measured by AUC in the gout and the control groups, and the box figure of MiG for all participants in the gout and the control groups were shown. (**C**) Accuracy of the microbiota-based model is measured by AUC in the validation group. (**D**) Accuracy of blood uric acid value based model is measured by AUC in the validation group.

For evaluation of gout disease based on these biomarkers, the correlation between the biomarkers and subjects were shown as a heat map, which indicated the ability of these biomarkers to discriminate healthy and gout hosts (Fig. 3A). Moreover, the Wilcoxon rank-sum test revealed that the MIGs between the patient group and the control group were significant (*P* < 0.05; Fig. 3B). Furthermore, performance of the MiG model in the training data (the 68-member cohort) was assessed by receiver operating characteristic curve (ROC). From the ROC of training dataset, we have determined the Youden index and the threshold of MiG as −2.157. For a given subject, a MiG that reaches this threshold would suggest increased risk of suffering gout. The area under the receiver operating characteristic (ROC) curves was 81.7% (Fig. 3B), indicating the microbial gout index could be used to classify gout individuals at high accuracy.

To validate the performance of this microbial model, an additional 15 subjects were included as the validation group. The blood uric acid values of these 15 subjects were higher than those of the control group but lower than that of severe gout patients (Supplementary Table 1). Thus uric acid value alone was not able to reliably diagnose gout for these individuals. Subsequent return-visits to clinics (six months from the initial diagnosis) confirmed that 6 subjects in this group suffered gout disease and 9 individuals were healthy. Analysis of their intestinal microbiota revealed that the accuracy of diagnosis via the microbial index based predictive model in the validation group reached 88.9% (Fig. 3C), higher than that based on blood uric acid (71.3%; Fig. 3D).

To probe the potential link between the organismal biomarkers of gout and the various blood indexes, Spearman rank correlations between the abundance matrixes of microbial biomarkers and each of the blood indices was performed (Supplementary Fig. 3A). Interestingly, positive correlation was observed between the amounts of uric acid, bilirubin, glutamic-pyruvic transaminase (GPT) and glutamic-oxalacetic transaminase (GOT) in the blood and the genera enriched in gout patients (such as *Bacteroides, Holdemania, Anaerotruncus,* etc), revealing a number of potential bacteria taxa whose activities might be implicated in the development of gout (Supplementary Fig. 3A).

### Mechanisms underlying Microbial Index of Gout

**Figure 4:**
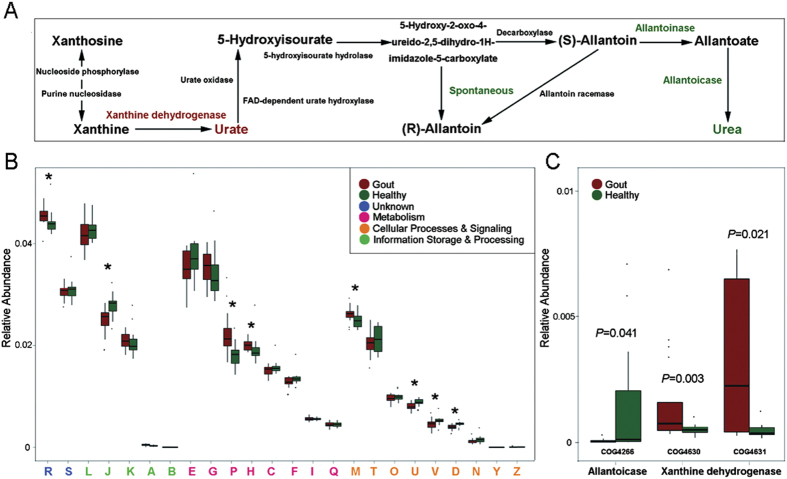
Functional features of gut microbiota in gout. (**A**) The metabolism of purine degradation. The enzymes in red were enriched in the gout patient group and those in green were enriched in the healthy (control) group. (**B**) Comparison of COGs between the patient and the control groups. A: RNA processing and modification; J: Translation, ribosomal structure and biogenesis; D: Cell cycle control, cell division, chromosome partitioning; M: Cell wall/membrane/envelope biogenesis; U: Intracellular trafficking, secretion, and vesicular transport; V: Defence mechanisms; H: Coenzyme transport and metabolism; P: Inorganic ion transport and metabolism; R: General function prediction only. The capital letters in red represent those functions enriched in the gout patient group, while the capital letters in blue represent those functions enriched in the control group. (**C**) Comparison of distribution of COGs of xanthine dehydrogenase and allantoicase between the patient and the control groups.

To probe the potential mechanism underlying the Microbial Indices of Gout, the shotgun metagenomic data were analyzed at the functional and metabolic pathway level. The results revealed that 5,245 KOs and 2,286 COGs were either positively or negatively associated with gout. The gout-associated KOs were then mapped to the reference metabolism pathway, which revealed that the gout patients were enriched in the metabolism of purine, starch and sucrose, sphingolipid, alanine, aspartate and glutamate, retinol, porphyrin and chlorophyll (Supplementary Table 9). On the pathway of purine metabolism, the xanthine dehydrogenase which can degrade the purine to uric acid was enriched in the gout patients, whereas the allantoinase that degrades the uric acid to urea was depleted (Fig. 4A). Thus it is possible that in the intestinal microbiota of gout patients, a significant amount of purine were degraded to uric acid which however could not be further degraded to urea, leading to abnormal accumulation of uric acid in gout patients. In contrast, the KO enriched in the healthy group included those involved in butyric acid biosynthesis, inositol phosphate metabolism, propanoate metabolism, methane metabolism, glycerolipid metabolism, thiamine metabolism and nitrotoluene degradation (Supplementary Table 9).

Analysis of the functional feature as defined by COGs revealed that the xanthine dehydrogenase (COG4360 and COG4361) was significantly enriched (*P* < 0.05, Wilcoxon rank-sum test, Fig. 4C) in gout patients, but the allantoicase (COG4266) depleted. These results were consistent with those based on the metabolic pathway of purine degradation. Moreover, the COGs that were enriched in gout patients include those implicated in RNA processing and modification, Cell wall/membrane/envelope biogenesis, Coenzyme transport and metabolism and Inorganic ion transport and metabolism (Fig. 4B and Supplementary Table 10). In contrast, COGs of Translation, ribosomal structure and biogenesis, Cell cycle control/division, Intracellular trafficking/secretion/vesicular transport and Defence mechanisms were enriched in the healthy group.

## Discussion

In current clinical practice, the blood uric acid value is considered to be the reference index in clinical gout diagnosis, however it can be hysteretic. Our diagnosis model for gout based on metagenomic genera of intestinal microbiota sampled from stool has shown promise in improving the sensitivity of diagnosis, where the model based on 17 genera achieved 88.9% accuracy. In fact, patients in the validation group that could not be reliably diagnosed by blood uric acid value can be correctly diagnosed as gout using the microbial index. Therefore the MiG may potentially be a valuable tool for early detection of gout.

The MiG revealed the presence of bacterial dysbiosis in gout patients. In the network of MGS, *Bacteroides caccae* (*n* = 7) and *Bacteroides xylanisolvens* (*n* = 4) were enriched in gout patients. In past studies, *Bacteroides caccae* was recognized one biomarker of IBD, and the *Omp*W protein produced by *B. caccae* was a target of the IBD-associated immune response^21^, thus the enriched intestinal *B. caccae* in gout patients could potentially induce serious inflammatory response. *Bacteroides xylanisolvens* strains isolated from feces of human and other animals was considered to have no virulence and be safe for food use^22^,^23^. Significant depletion of *Faecalibacterium prausnitzii* which was reported to have anti-inflammatory properties and contribute to gut health through butyrate production^24^ was observed for gout patients, potentially explaining the decline in butyric acid biosynthesis in gout patients. Protective mechanisms of butyric acid in human intestine include providing the nutrition for intestinal mucosa, promoting the growth and repairing of intestinal villus, enhancing the intestinal immunity, facilitating the growth of beneficial microbes and inhibiting the pathogenic bacteria^25^, thus decline in butyric acid biosynthesis can cause a number of physiological disorders in the host. On the other hand, the microbial xanthine dehydrogenase which can degrade the purine to uric acid was enriched in the gout patients whereas the microbial allantoinase that degrades the uric acid to the urea was depleted in gut patients. Thus, another possible link between activity of intestinal microbiota and gout pathogenesis might be that the overly abundant xanthine dehydrogenase and the relative deficiency of allantoinase in the intestinal microbiota might have accumulated more uric acid and consequently aggravated the gout symptoms.

Microbial dysbiosis in intestinal microbiota for two other chronic metabolic disorders, liver cirrhosis^26^ and T2D^27^, were recently reported, also based on cohorts of Chinese adults. We found that a decreased level in microbial metabolism of butyrate biosynthesis was a shared feature of the three kinds of diseases^26^,^27^. A remarkable similarity of reduction of *Faecalibacterium prausnitzii* and inhibition of butyrate biosynthesis was observed in patients from all the three chronic diseases (Table 3). This observation highlights the potential of *Faecalibacterium prausnitzii* as an indicator of intestine health.

However, in terms of the organismal structure of intestinal microbiota, T2D and gout patients were more similar, while both quite distinct from that of liver cirrhosis (Supplementary Fig. 4), in that patients suffering from T2D and gout diseases exhibit a lack of the species *Faecalibacterium prausnitzii, Roseburia intestinalis* and *Eubacterium rectale* (when compared with healthy individuals). Moreover, certain host features affected by intestinal microbiota similar to that observed for gout patients was also present in patients of T2D (Table 3 and Supplementary Fig. 4)^27^,^28^. In T2D patients, blood uric acid value are usually elevated. In fact, the diabetogenic effect of uric acid has been reported since 1950^29^, and here a positive correlation between blood uric acid and glucose values was also observed (Supplementary Fig. 3B). Moreover, many believed T2D as one serious complication of gout, and even considered the uric acid value as one indirect index for T2D diagnosis^30^. However, the etiological link between uric acid and T2D is not yet fully understood, although it was reported that uric acid itself might contribute to the exacerbation of insulin resistance^31^. Our results here suggested that a similar or shared functional disorder of intestinal microbiota might underlie the pathological link between the two metabolic syndromes.

Catalogs of reference genes in the human gut microbiome are crucial for functional metagenomic analyses in healthy and diseased populations. A large catalog of reference genes from intestinal microbiota that included 9,879,896 genes was recently reported^32^, however it was mainly derived from healthy subjects from MetaHIT and HMP, which might limit its reference value in help interpreting diseased microbiota. This point was supported by a recent study linking dysbiosis of intestinal microbiota to liver cirrhosis which concluded that the number of unique genes among gene sets of different disease, genotypes, ages and dietary habits can be large^26^. Thus the intestinal gene catalogue of gout established in this study that includes 2,653,431 microbial genes from both gout and healthy cohorts should be of value for future studies probing the role of intestinal microbiota in gout and related disorders.

The strong association between individual bacterial taxa and gout revealed here suggested the importance of including intestinal microbiota metabolism into the diagnosis and mechanistic interrogation of gout. For example, examination of the link between microbiota activity with host genetic disorders, such as dysfunction of human ABCG2 (a high-capacity urate transporter regulating serum uric acid levels, which was identified as one major reason for early-onset gout^33^), might reveal the interplay between microbiota and hosts in disease initiation and development. Moreover, tracking the structure and functional activity of intestinal microbiota in longitudinal studies in host populations of large size or from distinct ethical background or diet pattern should reveal the degree and nature of heterogeneity in microbiota among host individuals and promise to provide new ways of gout prevention and control.

## Materials and Methods

### Study design and subject recruitment

The study cohort was composed of three groups. Group one (case group) consisted of 35 gout patients, aged 32–75 years, and they were recruited from the First Affiliated Hospital of Baotou Medical College, located in Baotou, Inner Mongolia Autonomous Region of China. Diagnosis of gout was confirmed by the analysis of blood uric acid among patients claiming of painful joints. The second group (control group) consisted of 33 healthy individuals, aged 28–70 years. The validation group consisted of 15 subjects that were aged 28–69 years, among whom were 6 gout patients and 9 healthy individuals. When recruiting subjects for test group, we chose individuals with blood uric acid value higher than control group level but lower than serious gout patients’ level. All the subjects were asked to fill up a dietary diary, which recorded information such as their gender, age and diet for a period of three consecutive days prior to the collection of fecal samples (Supplementary Table 1). The study was approved by the Ethical Committee of the First Affiliated Hospital of Baotou Medical College (Baotou, China), and informed consent was obtained from all the 83 volunteers before enrollment in the study. Sampling and all subsequent steps described in the Materials and Methods have been conducted in accordance with the approved guidelines.

Fecal samples were collected from each individual in the morning before the first meal. After the weight of the fecal sample was determined, RNAlater® solution (Ambion, USA) was added into the tube in the ratio of one part fecal sample to five parts RNAlater® solution and mixed homogenously via vortexing and stored at −20 °C until further processing. All of the 83 samples were used for bacterial 16S rRNA genes V1-V3 region pyrosequencing and 39 samples (include 17 gout patients, 18 control individuals and 5 test individuals) were selected for whole-genome shotgun (WGS) sequencing.

### DNA extraction

The QIAamp® DNA Stool Mini Kit (Qiagen, Hilden, Germany) was used for DNA extraction from the fecal samples. The quality of the extracted DNA was assessed by 0.8% agarose gel electrophoresis, and the OD 260/280 was measured by spectrophotometry. All of the DNA samples were stored at −20 °C until further processing.

### PCR amplification of the bacterial 16S rRNA genes V1-V3 region and pyrosequencing

The V1-V3 region of the 16S ribosomal RNA (rRNA) genes was amplified for barcoded pyrosequencing for all 83 volunteers. A set of 10-nucleotide barcodes was added to the universal forward primer 27F (5′- AGAGTTTGATCCTGGCTCAG -3′) and the reverse primer 533R (5′-TTACCGCGGCTGCTGGCAC -3′), which were targeted at the domains Bacteria. PCR amplification was then performed as described previously^34^.

Quality of the PCR products was ensured using Agilent 2100 Bioanalyzer (Agilent Technologies, Palo Alto, Calif.) in accordance with the manufacturer’s instructions. The PCR products were pooled in equimolar ratios with a final concentration of 100 nmol/L for pyrosequencing (Roche GS FLX) performed by Shanghai Majorbio Bio-pharm Technology Co.,Ltd.

### Pyrosequencing sequence processing and bioinfomatics analysis

High-quality sequences were extracted from the raw reads and the extracted sequences were sorted into different samples according to the barcodes. After the removal of the barcodes and primer, the extracted sequences were processed mainly using the QIIME (v1.5.0) suite of software tools^35^.

By pyrosequencing, we generated a dataset consisting of 535,153 high-quality 16S rRNA gene sequences with an average of 6,398 sequences (range = 3,817–9,983, SD = 1,433) obtained for each of the 83 samples (Supplementary Table 2). Operational taxonomic units (OTUs) were delineated at a 97% similarity level, leaving 3,685 for further analysis.

### Whole-genome shotgun (WGS) sequencing and quality control

All samples were sequenced in the Illumina HiSeq2000 instrument. Libraries were prepared with a fragment length of approximately 300 bp. Paired-end reads were generated with 100 bp in the forward and reverse directions. The length of each read was trimmed with Sickle. Reads that aligned to the human genome were also removed. This set of high-quality reads was then used for further analysis. An average of 9.52 gigabases (Gb) paired-end reads were obtained for each sample, totaling 371.2 Gb of high-quality data that were free of human DNA and adaptor contaminants (Supplementary Table 3).

### Illumina short reads *de novo* assembly, gene prediction and construction of the non-redundant gene catalogue

The Illumina reads were assembled into contigs using IDBA-UD^36^ with default parameters. Genes were predicted on the contigs with MetaGeneMark^37^. A non-redundant gene catalogue was constructed with CD-HIT^38^ using a sequence indentity cut-off of 0.95, with a minimum coverage cut-off of 0.9 for the shorter sequences. This catalogue contained 2,653,431 microbial genes.

### Functional annotation

We aligned putative amino acid sequences, which were translated from the gene catalogue, against the proteins/domains in COG and KEGG databases using blastp (e-value ≤1e-5 with a bit-score higher than 60). Each protein was assigned to the KEGG orthologue group (KO) or cluster of orthologous group (COG) by the highest scoring annotated hit.

### Computation of relative gene abundance

To assess the abundance of genes, reads were aligned to the gene catalogue with Bowtie2^39^ using parameters: -p 12 -x nt -1 R1.fastq -2 R2.fastq -S R.sam. Then, for any sample N, we calculated the abundance as follows:

Step 1: Calculation of the copy number of each gene:


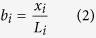


Step 2: Calculation of the relative abundance of gene i


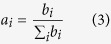


*a*_*i*_: the relative abundance of gene i

*b*_*i*_: the copy number of gene i from sample N

*L*_*i*_: the length of gene i

*x*_*i*_: the number of mapped reads

### Metagenomic species (MGS)

The co-abundance principle and canopy clustering algorithm were applied to generate the metagenomic species (MGS). The detailed approach was described in previous references^20^,^26^. MGS were assigned to a given genome when more than 80% of its sub-gene matched the same genome using blastN, at a threshold of 95% identity over 90% of gene length. Those MGS that cannot meet the above conditions were annotated using blastp analysis. If over 80% of the genes from a MGS had the same taxonomical level of assignment, this MGS was identified as the organism.

### Statistical analysis

All statistical analyses were made in the R software. We performed the PERMANOVA^40^ (Permutational multivariate analysis of variance) analysis using the method implemented in R package “vegan”, and the permuted *P*-value was obtained by 10,000 times permutations. PCA and Procuste analysis was performed in R using the ade4^41^ package. Differential abundance of genus, genes and COGs were tested by Wilcoxon rank sum test, and the significant different (*p* < 0.01) genus, genes and COGs were considered as the potential gout biomarkers. We applied the Receiver Operator Characteristic (ROC) analysis to assess the performance of the gout metagenomic biomarkers by using the “pROC” package in R software^42^. The correlation between biomarkers and blood index were calculated by Spearman rank correlation coefficient and visualized by heatmap in R using the “pheatmap” package.

## Additional Information

**Data Availability:** The sequence data reported in this paper have been deposited in the MG-RAST database (Project No. 9870).

**How to cite this article**: Guo, Z. *et al.* Intestinal Microbiota Distinguish Gout Patients from Healthy Humans. *Sci. Rep.* **6**, 20602; doi: 10.1038/srep20602 (2016).

## Supplementary Material

Supplementary Information

Supplementary Dataset

## Figures and Tables

**Table 1:** Sample information and blood index.

**Group**	**Subjects**	**Age**	**Male**	**Num. with 16S rRNA amplicon sequencing**	**Average reads**	**Num. with WGS sequencing**	**Average num. of reads**
Gout	*n* = 35	50.31 ± 10.16	19/35	35/35	6107	16/35	59117661
Control	*n* = 33	48.73 ± 11.59	19/33	33/33	6594	18/33	59657047
Validation	*n* = 15	46.87 ± 11.46	10/15	15/15	6670	5/15	61574689
**Group**	**BMI**	Uric Acid*	Total Bilirubin*	GPT*	GOT*	**ALP**	**Total Protein**
Gout	23.09±3.48	510.94 ± 91.47	14.92 ± 6.29	30.43 ± 9.75	24.31 ± 10.03	57.74 ± 11.81	67.97 ± 8.63
Control	22.93 ± 3.81	199.48 ± 67.14	9.16 ± 3.78	13.73 ± 5.18	11.55 ± 5.43	59.42 ± 14.18	69.79 ± 5.48
Validation	23.99 ± 3.47	402.73 ± 58.38	13.15 ± 4.67	22.93 ± 9.92	25.27 ± 6.38	67.40 ± 15.90	72.97 ± 8.47
**Group**	Urea Nitrogen*	**Creatinine**	**Blood Glucose**	**Triglyceride**	**Cholesterol**	**HDLC**	**LDLC**
Gout	7.49 ± 3.12	81.37 ± 20.61	5.73 ± 1.49	1.51 ± 0.64	4.61 ± 1.17	1.20 ± 0.19	2.69 ± 0.96
Control	4.53 ± 1.22	82.54 ± 13.71	5.12 ± 0.74	1.45 ± 0.59	4.74 ± 1.01	1.17 ± 0.28	2.67 ± 0.74
Validation	6.37 ± 4.36	78.78 ± 12.19	5.59 ± 1.01	1.35 ± 0.28	4.72 ± 0.85	1.20 ± 0.21	2.82 ± 0.51

NOTE: GPT represent glutamic-pyruvic transamine; GOT represent glutamic-oxalacetic transaminase; HDLC represent high-density lipoprotein cholesterol; LDLC represent low-density lipoprotein cholesterol.

**Table 2:** Microbial biomarkers of the gout disease.

**Genus**	***P*** **values (adjusted)**	**Odds ratios**	**Enriched**	**Genus**	***P*** **values (adjusted)**	**Odds ratios**	**Enriched**
*Coprococcus*	0.0005231	2.19	Control	*Barnesiella*	0.0041493	0.22	Gout
*Faecalibacterium*	0.0022616	9.62	Control	*Parasporobacterium*	0.0054789	0.06	Gout
*Oscillibacter*	0.0024974	1.00	Control	*Paraprevotella*	0.0093753	0.05	Gout
*Ruminococcus*	0.0064096	1.28	Control	*Anaerotruncus*	0.0106759	0.05	Gout
*Odoribacter*	0.0115741	0.17	Control	*Pseudobutyrivibrio*	0.0319377	0.19	Gout
*Subdoligranulum*	0.0210841	2.56	Control	*Bacteroides*	0.0368476	49.67	Gout
*Robinsoniella*	0.0286258	0.01	Control	*Holdemania*	0.0431658	0.02	Gout
*Dialister*	0.0325178	0.38	Control	*Acetanaerobacterium*	0.047967	0.01	Gout
*Alistipes*	0.0360435	0.77	Control				

**Table 3:** Comparison of taxonomy and functional features in different chronic diseases.

	**Metagenomic Species**	**T2D**	**Gout**	**LC**		**Metagenomic Species**	**T2D**	**Gout**	**LC**
Enriched species in T2D/Gout/LC	*Akkermansia muciniphila*	+	n/a	n/a	Depleted species in T2D/Gout/LC	*Faecalibacterium prausnitzii*	−	−	−
	*Bacteroides intestinalis*	+	n/a	n/a		*Haemophilus parainfluenzae*	−	n/a	n/a
	*Clostridium bolteae*	+	+	n/a		*Roseburia intestinalis*	−	−	n/a
	*Clostridium hathewayi*	+	n/a	n/a		*Roseburia inulinivorans*	−	n/a	n/a
	*Clostridium ramosum*	+	n/a	n/a		*Eubacterium rectale*	−	−	n/a
	*Clostridium symbiosum*	+	n/a	n/a		*Bacteroides stercoris*	n/a	−	n/a
	*Eggerthella lenta*	+	n/a	n/a		*Bifidobacterium dentium*	n/a	−	n/a
	*Escherichia coli*	+	n/a	n/a		*Bifidobacterium pseudocatenulatum*	n/a	−	n/a
	*Bacteroides caccae*	n/a	+	n/a		*Clostridium bolteae*	n/a	−	n/a
	*Bacteroides xylanisolvens*	n/a	+	n/a		*Coprococcus catus*	n/a	−	n/a
	*Bacteroides ovatus*	n/a	+	n/a		*Dorea formicigenerans*	n/a	−	n/a
	*Eubacterium rectale*	n/a	+	n/a		*Bacteroides plebeius*	n/a	−	n/a
	*Lactobacillus fermentum*	n/a	n/a	+		*Bacteroides clarus*	n/a	n/a	−
	*Lactobacillus mucosae*	n/a	n/a	+		*Bacteroides uniformis*	n/a	n/a	−
	*Lactobacillus salivarius*	n/a	n/a	+		*Bilophila wadsworthia*	n/a	n/a	−
	*Veillonella atypica*	n/a	n/a	+		*Coprococcus comes*	n/a	n/a	−
	*Veillonella dispar*	n/a	n/a	+	Depleted Pathway in T2D/Gout/LC	Metabolic Pathway	T2D	Gout	LC
	*Veillonella parvula*	n/a	n/a	+		Butyrate biosynthesis	−	−	−
	*Megasphaera micronuciformis*	n/a	n/a	+		Cell motility	−	n/a	n/a
	*Prevotella buccae*	n/a	n/a	+		Cofactors & vitamins Metabolism	−	n/a	n/a
	*Clostridium perfringens*	n/a	n/a	+		CH4 metabolism	n/a	−	n/a
	*Ruminococcus gnavus*	n/a	n/a	+		Amino acid metabolism	n/a	n/a	−
	*Streptococcus anginosus*	n/a	n/a	+		Carbohydrate metabolism	n/a	n/a	−
	*Streptococcus oralis*	n/a	n/a	+		Energy metabolism	n/a	n/a	−
	*Streptococcus salivarius*	n/a	n/a	+		Signal transduction	n/a	n/a	−
