# Supplementary material for: Intestinal Microbiota Distinguish Gout Patients from Healthy Humans
Source: Sci Rep. 2016 Feb 8;6:20602. doi: 10.1038/srep20602 (PMC4757479; doi:10.1038/srep20602)
Supplement: Supplementary Information [file srep20602-s1.pdf]

Supplementary Tables and Figures

Intestinal microbiota distinguish gout patients from healthy humans

Zhuang Guo[1]†, Jiachao Zhang[1]†, Zhanli Wang[3]†, Kay Ying Ang[2]†, Shi Huang[4], Qiangchuan Hou[1], Xiaoquan Su[4], Jianmin Qiao[1], Yi Zheng[1], Lifeng Wang[1], Eileen Koh[2], Ho Danliang[2], Jian Xu[4], Yuan Kun Lee[2], Heping Zhang[1]

The supplementary imformation includes:

Supplementary Figures S1-S4

Supplementary Figures

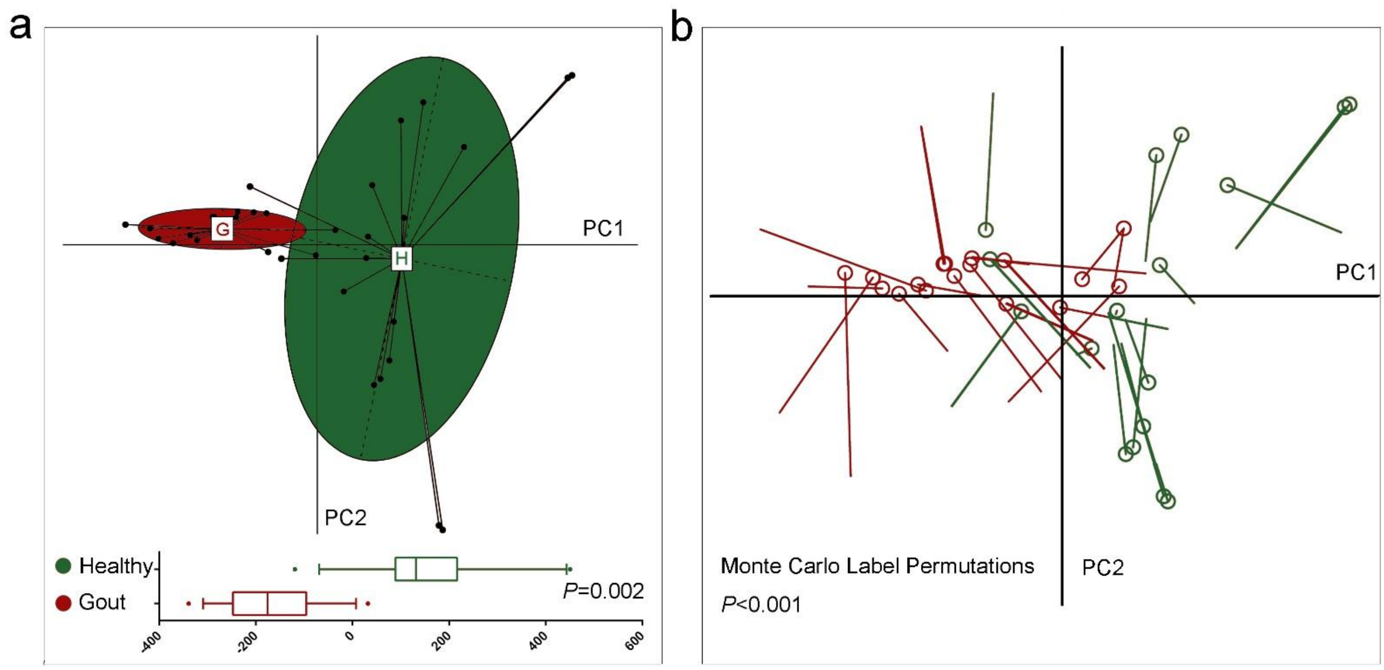

Figure S1. The functional distinction of intestinal microbiota between gout patients and healthy controls. (A) Principal Component Analysis (PCA) based on microbial gene profile. The red and black points represented the gout-active and healthy individuals respectively. (B) Procrustes analysis revealed that human gut bacterial lineages and microbiome COGs content gave similar clustering patterns. The cycle end of each line represented the 16S rRNA data for the sample, whereas the other end represented to the functional annotation of that particular sample. The fit of each Procrustes transformation over the first four dimensions was reported as the *P*-value by 10, 000 Monte Carlo label permutations.

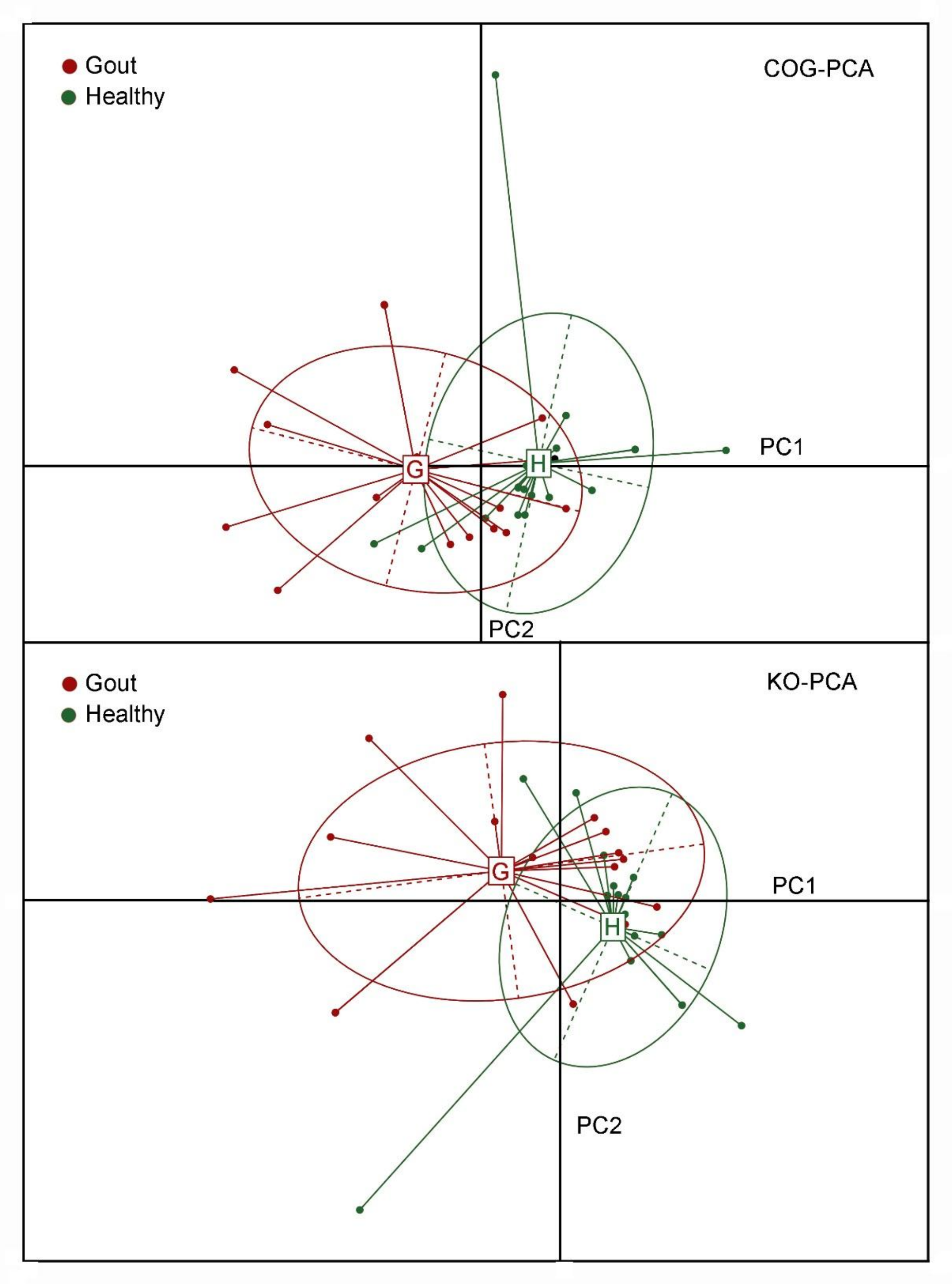

Figure S2. PCA results of COG and KO profiles. Points in red represented the gout patients and points in green represented the healthy individuals.

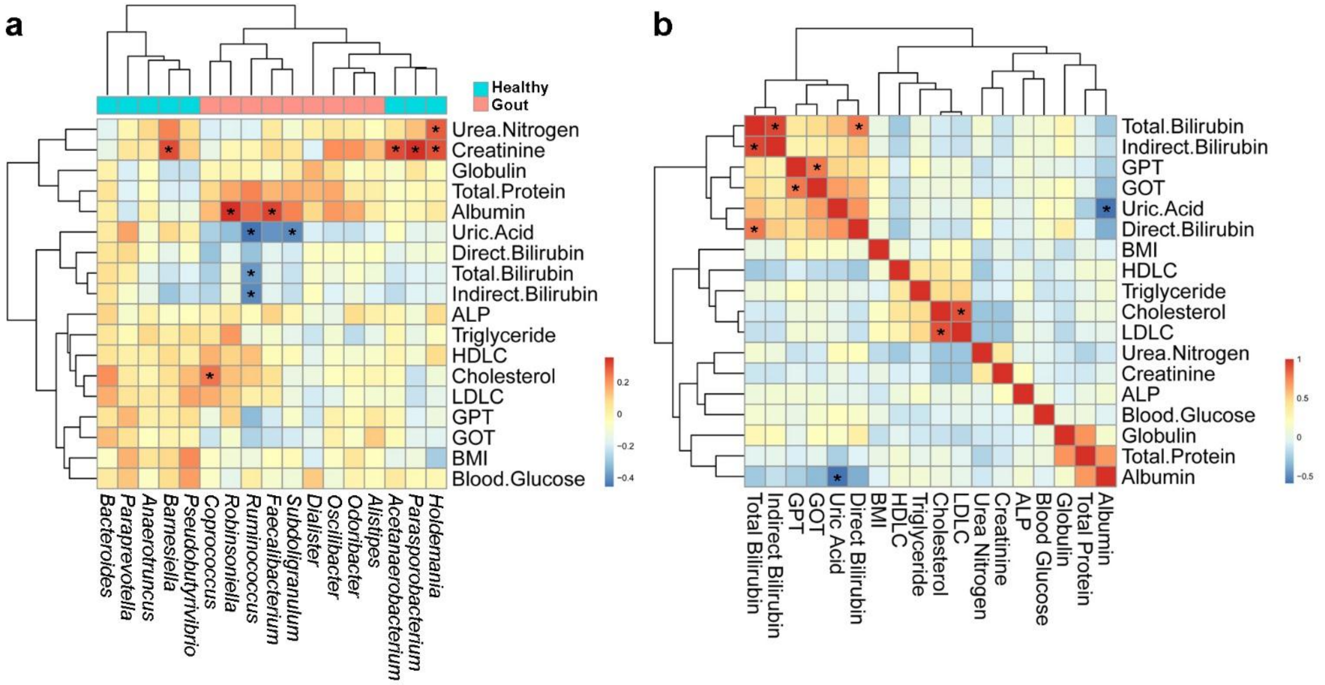

Figure S3. Correlation between the microbial biomarkers of gout and the various blood indices. Spearman's rank correlation coefficients and *P* values ("*" represented the *P* <0.05) for the correlation were shown.

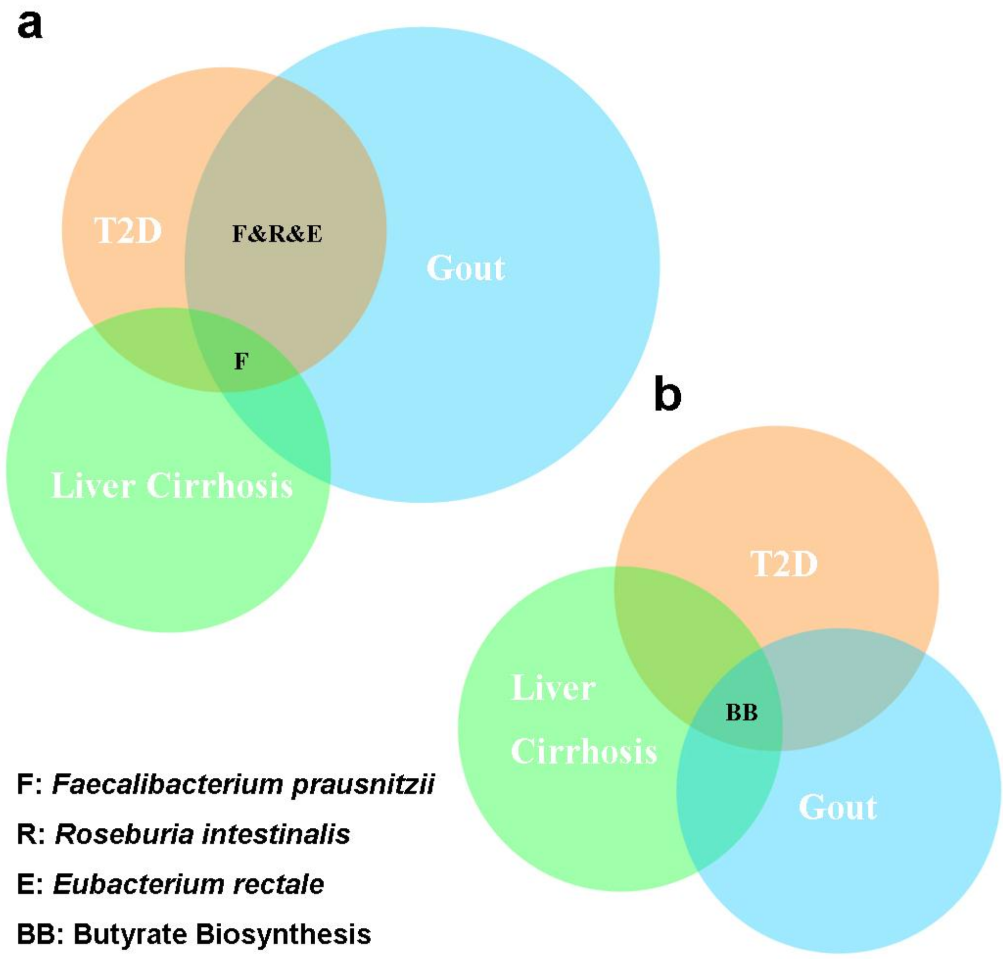

Figure S4. Comparison between microbial signatures of gout and those of T2D and liver cirrhosis. The depletion of *Faecalibacterium prausnitzii* and inhibition of butyrate biosynthesis were shared among patients from all the three chronic diseases**.**
